# Ablation of RIC8A Function in Mouse Neurons Leads to a Severe Neuromuscular Phenotype and Postnatal Death

**DOI:** 10.1371/journal.pone.0074031

**Published:** 2013-08-16

**Authors:** Katrin Ruisu, Keiu Kask, Riho Meier, Merly Saare, Raivo Raid, Alar Veraksitš, Alar Karis, Tambet Tõnissoo, Margus Pooga

**Affiliations:** 1 Institute of Molecular and Cell Biology, University of Tartu, Tartu, Estonia; 2 Institute of Biomedicine and Translational Medicine, Department of Physiology, University of Tartu, Tartu, Estonia; IGBMC/ICS, France

## Abstract

Resistance to inhibitors of cholinesterase 8 (RIC8) is a guanine nucleotide exchange factor required for the intracellular regulation of G protein signalling. RIC8 activates different Gα subunits via non-canonical pathway, thereby amplifying and prolonging the G protein mediated signal. In order to circumvent the embryonic lethality associated with the absence of RIC8A and to study its role in the nervous system, we constructed *Ric8a* conditional knockout mice using Cre/loxP technology. Introduction of a synapsin I promoter driven Cre transgenic mouse strain (*SynCre*) into the floxed *Ric8a* (*Ric8a* ^*F/F*^) background ablated RIC8A function in most differentiated neuron populations. Mutant *SynCre* ^*+/-*^ *Ric8* ^*lacZ/F*^ mice were born at expected Mendelian ratio, but they died in early postnatal age (P4-P6). The mutants exhibited major developmental defects, like growth retardation and muscular weakness, impaired coordination and balance, muscular spasms and abnormal heart beat. Histological analysis revealed that the deficiency of RIC8A in neurons caused skeletal muscle atrophy and heart muscle hypoplasia, in addition, the sinoatrial node was misplaced and its size reduced. However, we did not observe gross morphological changes in brains of *SynCre* ^*+/-*^ *Ric8a* ^*lacZ/F*^ mutants. Our results demonstrate that in mice the activity of RIC8A in neurons is essential for survival and its deficiency causes a severe neuromuscular phenotype.

## Introduction

Heterotrimeric G proteins receive signals from ligand-bound G protein-coupled receptors (GPCRs) to activate a cascade of downstream responses that finally cause changes in the cell physiology [1]. In addition to receptors, multiple modulatory proteins are known to regulate the G protein activation [2–4]. One of these non-receptor activators of G proteins is Resistance to Inhibitors of Cholinesterase 8 (RIC8), a conserved guanine nucleotide exchange factor (GEF) for subset of Gα subunits. In mammals two *Ric8* genes have been identified: *Ric8a* and *Ric8b*. RIC8A is able to interact with Gα_q_, Gα_i1_, Gα_o_, Gα_12/13_ but not Gα_s_ *in vitro*, whereas RIC8B has been shown to interact with Gα_s_, Gα_q_, Gα_olf_ and Gα_13_ [2,5–7]. RIC8A forms a complex with inactive Gα proteins bound to guanosine diphosphate (GDP), stimulating the release of GDP and stabilizing nucleotide-free Gα subunit. The binding of guanosine triphosphate (GTP) to Gα disrupts the complex, RIC8A disassociates and activated Gα is released [2]. RIC8A has been demonstrated to play a critical role in various G protein mediated cellular processes such as cell division, migration and synaptic signalling [8–16]. Recently, an additional role for RIC8A has been suggested as a molecular chaperone that regulates G protein biosynthesis [17,18].

The function of RIC8A in neuronal cells or nervous system of mammals has not yet been sufficiently analysed. Several studies with other mammalian cells and tissues corroborate the results of the biochemical studies and support the function of RIC8A as a signal amplifier for Gα subunits [19]. The expression pattern of *Ric8a* in the early stages of mouse organogenesis (E9.5-E12.0) is highly neurospecific [20]. Additionally, *Ric8a* is expressed in several areas of the adult mouse brain (e.g. in the neocortex, cingulate cortex, caudate putamen, hippocampus, cerebellum), which are important for the regulation of mouse behaviour [20,21]. Moreover, *Ric8a* haploinsufficiency results in behavioural abnormalities, such as increased anxiety-like behaviour and impaired spatial memory as demonstrated in heterozygous *Ric8a* ^*+/-*^ mice [21]. Homozygous *Ric8a* ^*-/-*^ mouse embryos display multiple gastrulation defects, which lead to embryonic lethality at E6.5 – E9.5 [17,22].

In order to circumvent the embryonic lethality and investigate the role of RIC8A in the mouse nervous system, we generated conditional knockout mice where RIC8A was specifically knocked out in postmitotic neurons. For this we used transgenic mice expressing Cre-recombinase under *Synapsin I* promoter (*SynCre*) utilised in many laboratories [23–25]. Introduction of a *SynCre* transgenic mouse strain into the conditional *Ric8a* (*Ric8a* ^*lacZ/F*^) background ablated RIC8A function in differentiated neurons. In this study, we analysed the neuromuscular phenotype of such mice and provided novel information about the vital role of RIC8A in mammalian nervous system.

## Materials and Methods

### Animals

All the mice used in this research had isogenic C57BL/6J background. A targeting vector was designed to insert a single loxP site upstream of exon 1 into the XhoI site and a loxP-flanked neomycin cassette downstream of exon 4 into the Bsp120I site. The construct was electroporated into the E14.1 embryonic stem (ES) cells form 129/Ola mice [26]. Correctly targeted ES cells were transiently transfected with a Cre-recombinase expressing plasmid to remove the neomycin cassette, leaving exons 1-4 flanked by loxP sites (*Ric8a* ^*F/F*^). The ES cell derivatives with two loxP sites were injected into C57BL/6J blastocysts, which were injected into pseudopregnant foster females. The resulting chimeric mice were bred to C57BL/6J mice. To achieve deletion of *Ric8a* specifically in differentiated neurons, we crossed *SynCre* mice (B6.Cg-Tg(Syn1-cre) 671Jxm/J) [23] with *Ric8a* ^lacZ/+^ mice, whose first 5 exons of one allele of *Ric8a* gene were replaced with β-geo cassette [21] to create *SynCre* ^*+/-*^ *Ric8a* ^*lacZ/+*^ mice. Male *SynCre* ^*+/-*^ *Ric8a* ^*lacZ/+*^ mice were crossed with *Ric8a* ^F/F^ female mice to produce *SynCre* ^*+/-*^ *Ric8a* ^lacZ/F^ conditional knockout mutants (from now on termed as *Ric8a* ^*CKO*^). Littermate controls that were used in this report are a pool of phenotypically indistinguishable mice with three genotypes: *SynCre* ^*+/-*^ *Ric8a* ^*F/F*^, *SynCre* ^*-/-*^ *Ric8a* ^*lacZ/F*^, *SynCre* ^*-/-*^ *Ric8a* ^*F/F*^ (from now on termed as littermate controls). To evaluate the specificity of *SynCre* expression, *SynCre* transgenic mice were crossed with Cre-dependent *lacZ* reporter line R26R (B6; 129-Gt(*ROSA*) *26Sor* ^*tm1Sho*^/J) [27]. Research was conducted with mice at P0 - P5 and E12.5 embryos were used for one experiment. The animals were maintained under a 12-hour light/dark cycle, with food and water available *ad libitum*. All experiments were performed in accordance with Directive of the Council of the European Communities (86/609/EEC) and approved by Estonian National Board of Animal Experiments.

### Genotyping

Genotypes of *Ric8a* ^*CKO*^ mutants were determined by polymerase chain reaction (PCR) using tail DNA with separate pairs of primers for detecting the presence of each transgenic allele: *SynCre* (GTCCAGACCCTACGGACAAG and CTAATCGCCATCTTCCAGCAGG), *Ric8a* ^*F*^ (GGTAGGGCTCAATGTTGG and GCCAAACAATCTCTCGAACC) and *Ric8a* ^*lacZ*^ (CGCATCGTAACCGTGCATCT, CTCTCCCAGCATCCCTCAC and CACACCCCAGCCGAGTTG). The functioning of Cre-recombinase in *Ric8a* ^*CKO*^ mutant mice was assessed using following primers CTTTTCCACGGGTGTTCTTC and GCCAAACAATCTCTCGAACC (all given primers are from 5´ to 3´).

### Quantitative real-time-PCR

Total RNA was purified from dissected brain structures and other tissues of *Ric8a* ^*CKO*^ mice and littermate controls by using Trizol^®^ Reagent (Life Technologies, Carlsbad, CA, USA), and cDNA was synthesized from the RNA with SuperScript^TM^ First-Strand Synthesis System (Life Technologies) using the manufacturer’s protocol. Quantitative RT-PCR (using Life Technologies Applied Biosystems StepOnePlus Real-Time PCR instrument) was performed using 1 µg of cDNA of respective tissues. The reaction was carried out for 40 cycles of 15 seconds at 95°C and 1 minute at 60°C in qPCR SyberGreen Mastermix (HOT FIREPol® EvaGreen® qPCR Mix Plus [ROX], Solis BioDyne, Estonia). Three independent experiments were performed for each sample. Constitutively expressed housekeeping gene *Hprt* (Hypoxanthine-guanine phosphoribosyltransferase) was chosen as reference using 5′-CACAGGACTAGAACACCTGC-3′ and 5′-GCTGGTGAAAAGGACCTCT-3′ primers. For relative *Ric8a* mRNA expression analysis primers 5′-GAGGAGTTCCACGGCCACA-3′ and 5′-CTTCAGCCTGTGGGTCTGGTG-3′ were used.

### In situ hybridization

The *in situ* hybridization analysis was conducted with freely floating brain sections. *Ric8a* riboprobe was transcribed *in vitro* from linearized plasmid that contains *Ric8a* cDNA fragment against first 478 bp (containing exons 1, 2 and a part of exon 3 until NheI site) using digoxigenin-labeled UTP (Roche Diagnostics GmbH) and T3 or T7 RNA polymerase (Roche Diagnostics GmbH). Dissected brains were fixed in 4% paraformaldehyde (PFA) overnight, cryoprotected and sectioned in a coronal plane at thickness of 40 µm. Free floating sections were rinsed with phosphate-buffered saline (PBS) for 5 minutes and incubated in hybridization solution [50% formamide, 5x saline-sodium citrate (SSC), 50 µg/ml heparin, 250 µg/ml herring sperm DNA, 2% blocking reagent (Roche Diagnostics GmbH)] at 65^°^C for 2 hours. The RNA probe (1 µg/ml) was added and hybridized at 65^°^C for 12–16 hours. After hybridization, sections were washed in 50% formamide, 5x SSC, 0.1% sodium dodecyl sulfate (SDS) at 65^°^C for 30 minutes and then twice in 50% formamide, 2x SSC at 65^°^C for 30 minutes and several times in Tris-buffered saline with Tween 20 (TBST). Sections were incubated in 2% blocking reagent at room temperature. Hybridization signal was detected using alkaline phosphatase-conjugated anti-digoxigenin antibody (1:2000; Roche Diagnostics GmbH) overnight at 4^°^C. Sections were washed several times in TBST and alkaline phosphatase buffer (100 mM Tris-HCl pH 9.5, 100 mM NaCl, 20 mM MgCl_2_ 0.1% Tween 20, 2mM levamisole) before signal was developed with BM purple (Roche Diagnostics GmbH). Sections were rinsed several times with PBS and placed on slides covered with 0.5% gelatine, dried and mounted with DePeX (Serva Electrophoresis GmbH). Control sections were incubated in an identical concentration with the control sense probe. No staining was found in the control sections.

### Western blotting

Standard Western blotting procedures were performed using a Bio-Rad electrophoresis and transfer apparatus. Proteins were separated on 4–10% SDS-PAGE gradient gel and transferred onto polyvinylidene fluoride membrane (Millipore). Membrane was blocked with 10% non-fat dry milk and then incubated with mouse monoclonal antibody to RIC8A (Abcam). A mouse monoclonal antibody to GAPDH (GeneTex, Inc) was used as a control for protein loading. As secondary antibody, we used antibody against mouse IgG conjugated to alkaline phosphatase (Vector Laboratories). Staining was visualized in standard alkaline phosphatase buffer supplied with Nitrotetrazolium Blue chloride (Sigma-Aldrich) and 5-Bromo-4-chloro-3-indolyl phosphate disodium salt (Sigma-Aldrich).

### Whole mount X-Gal staining

*Ric8a* ^lacZ/+^ and *SynCre* ^*+/-*^ *R26R* embryos were dissected from the uterus in PBS and fixed for 2 hours in 4% PFA, rinsed twice with PBS, stained for 1 hour to overnight at 37°C in X-Gal buffer (1.3 mg/ml potassium ferrocyanide, 1 mg/ml potassium ferricyanide, 0.2% Triton X-100, 1 mM MgCl_2_, and 1 mg/ml X-Gal in PBS [pH 7.2]) and post-fixed. All embryos were cleared for better visualization: dehydrated through graded steps into 100% methanol and transferred into a solution of benzyl benzoate and benzyl alcohol (1:2) and photographed.

### Histological and anatomical analyses

P0 – P5 mice were weighed, measured, photographed and then sacrificed. Their organs were dissected, photographed and weighed. Excised organs were stored in 4% PFA for overnight at 4°C and processed for paraffin sectioning. Serial sections (8 µm) were prepared using Microm Ergostar HM200 (Microm International GmbH). Paraffin sections were stained by using hematoxylin and eosin as described previously [28] and mounted on glass slides into DePeX (Serva Electrophoresis GmbH). Hematoxylin-eosin stained muscle fibre cross sections were analysed using Cell^B image-acquisition software (Olympus). The number of motor neurons per section in 8 µm hematoxylin-eosin stained spinal cord cross sections was counted by experimenters blind to genotype. From each animal 90 sections (30 from each of the spinal cord regions, cervical, thoracic and lumbar) separated by at least 40 µm were analysed. Motor neurons were defined as large neuron somas with well-defined nucleus located in ventral horns.

### Electrocardiography

Heartbeat of conscious P1 *Ric8a* ^*CKO*^ mice and littermate controls [45-50 mm long and weighed approximately 1,47 g (*Ric8a* ^*CKO*^) -1,71 g (control)] was registered by EKG using PowerLab equipment. Commercially available EKG electrodes (Skintact® T-601) which contained viscous glue-gel were used and mice were placed on the electrodes on their left side to enable recordings close to II standard lead.

### Skeleton preparations

Mice at the age of P3 were dissected by removing the skin and organs and fixed in 96% ethanol for 48 hours for staining of skeleton. Cartilaginous parts of the bone were stained with 0,015% Alcian Blue 8GX (Sigma-Aldrich) in 1/5 (v/v) acetic acid/ethanol for about 7 days and then placed into 96% ethanol for 2 days. Tissues around the skeleton were cleared with 1% KOH for about 2-4 days and the clearing solution replaced with glycerol. Skeletons were stored in glycerol.

### Behavioural assessment

3 reflexes of neonatal mice were analysed: 1) “Pinching response” – tail or limbs of pups were lightly pinched with forceps. Reaction was estimated as “present” or “not”. 2) “Righting reflex” – pups were placed on their backs on a flat surface and monitored whether they could turn themselves over to rest in the normal position with all four feet on the ground in 30 seconds. 3) “Tail suspension test” – pups were suspended by the tail for 15 seconds and their hind- and forelimb posture was estimated. Hind limbs spread open and forelimbs grasping the air was registered as a normal response.

### Statistical analyses

Statistical analysis was performed using the Student’s *t* test (parametric analysis), and the results were expressed as mean ± SEM. A probability (P) value of less than 0.05 was considered statistically significant.

## Results

### Deletion of Ric8a in the mouse differentiated neurons

**Figure 1:**
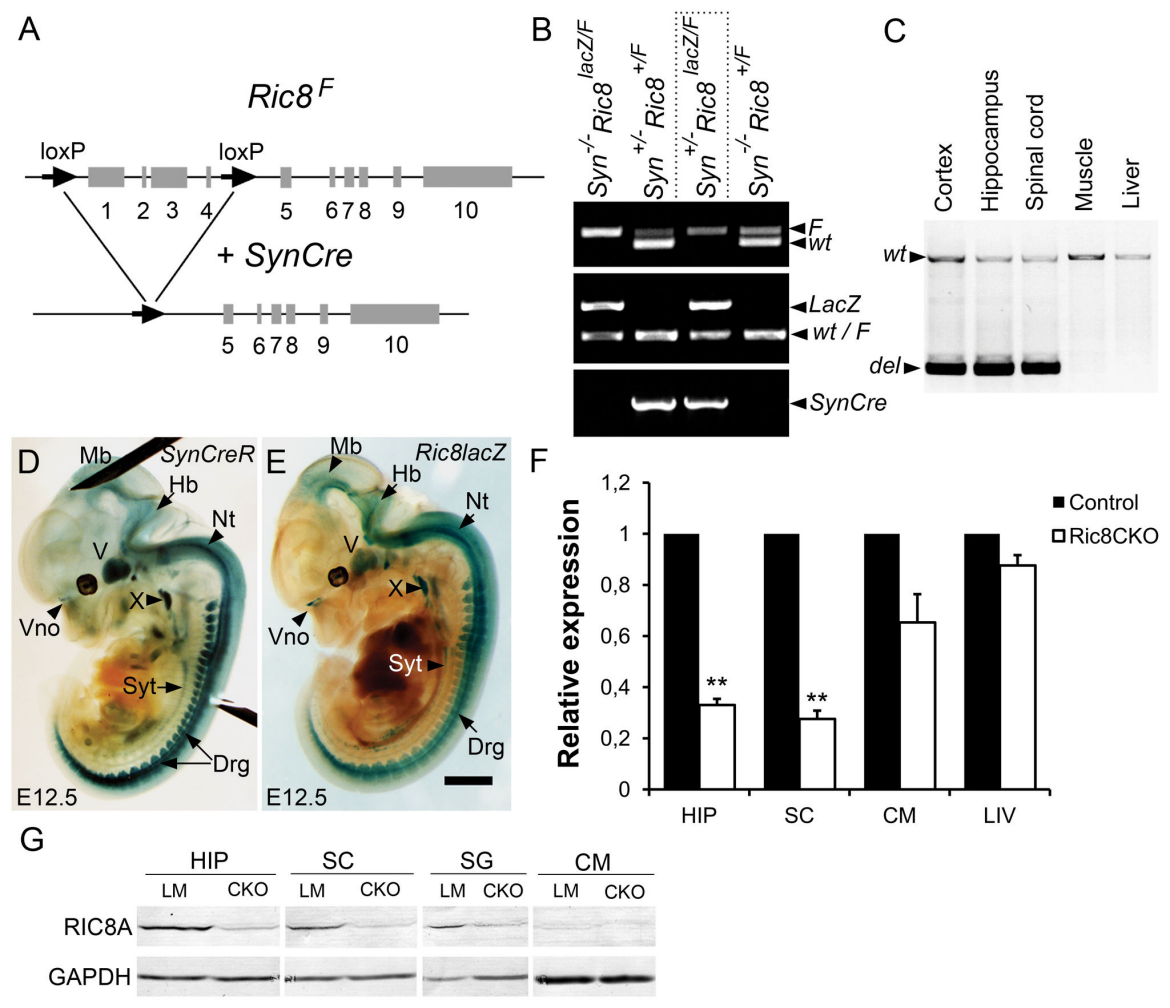
Neurospecific deletion of *Ric8a* in mice. (**A**) Schematic representation of neuron-specific deletion of the *Ric8a* gene. First four exons of floxed *Ric8a* are removed by Cre-recombinase which expression is under the control of *Synapsin I* promoter (SynCre). Numbered boxes represent *Ric8a* exons and arrows *loxP* sites. (**B**) PCR-based genotyping of mice using DNA from tail samples to detect *SynCre* transgene, floxed *Ric8a* allele and *LacZ* allele respectively. *Ric8a* ^*CKO*^ genotype is emphasized with dotted line. (**C**) Representative PCR showing the deletion of floxed *Ric8a* in *Ric8a* ^*CKO*^ mouse nervous system and no deletion in non-neural organs. (**D** and **E**) Comparison of Cre-recombinase (in *SynCre* ^*+/-*^ *R26R*) and *Ric8a* (in *Ric8a* ^*lacZ/+*^) expression in E12.5 embryos by X-gal staining. (**F**) Down regulation of *Ric8a* mRNA expression in *Ric8a* ^*CKO*^ mice relative to littermate control in hippocampus (HIP), spinal cord (SC), cardiac muscle (CM) and liver (LIV). (**G**) Deficiency of RIC8A protein in *Ric8a* ^*CKO*^ (CKO) mice compared to littermate control (LM) in hippocampus (HIP), spinal cord (SC), spinal ganglia (SG) and cardiac muscle (CM). GAPDH was used as a reference. Abbreviations: del, PCR fragment from the deleted allele Drg, dorsal root ganglia; F, floxed allele; Hb, hindbrain; Mb, midbrain; Nt, neural tube; SC, spinal cord; SG, spinal ganglia; Syt, sympathetic trunk; Vno, vomeronasal organ; wt, PCR fragment from the wild-type allele; V, trigeminal ganglion; X, vagus ganglion; ** *P* < 0,01. Error bars represent mean ± SEM scores. Scale bars: (D and E) 1 mm.

Since *Ric8a* deficient mice die during early development due to gastrulation defects [22] and haploinsufficiency of *Ric8a* causes behavioural abnormalities in mice [21], we used a conditional knockout approach to analyse the function of RIC8A in neural tissue. We produced neuron specific RIC8A deficient mice using Cre/LoxP technology: first, we generated *Ric8a* ^*F/F*^ flox mice with *loxP* sites flanking exons 1-4 of the *Ric8a* locus (Figure 1A). In order to characterize the *Ric8a* ^F/F^ mice in detail we crossed *Ric8a* ^F/F^ mice with X chromosome specific X-Cre transgenic mice (http://jaxmice.jax.org/strain/003465.html). As expected, the offspring from this crossing closely resemble *Ric8* ^*-/-*^ mutants and die during gastrulation as described earlier [21,22]. Homozygous *Ric8a* ^*F/F*^ mice were viable and fertile and had no evident morphological alterations. Next we crossed *Ric8a* ^F/F^ female mice with *SynCre* ^*+/-*^ *Ric8a* ^lacZ/+^ males (in which Cre-recombinase is expressed specifically in postmitotic neurons under the control of a Synapsin I promoter and the first five exons of one allele of *Ric8a* gene is replaced with β-geo cassette [21]) to produce *SynCre* ^*+/-*^ *Ric8a* ^lacZ/F^ (from now on named as *Ric8a* ^*CKO*^) conditional knockout mutants (Figure 1A). We affirmed the genotype of the produced mutants (Figure 1B, marked with dotted line) by allele specific PCR. Littermates with other genotypes had no phenotypic effect and thus we used them in this study as controls (Figure 1B). The specificity of *SynCre* expression was verified beforehand by crossing *SynCre* transgenic mice with Cre-dependent *lacZ* reporter line R26R [27]. It has been shown earlier that *SynCre* expression starts at E12.5, and it is restricted to brain, spinal cord and dorsal root ganglia (DRG) [23]. However, our experiments revealed more extensive expression of *SynCre* at E12.5, we also observed β-galactosidase activity in the sympathetic trunk, cranial ganglia, vomeronasal organ and eye (Figure 1D). The activity pattern of *SynCre* coincided very well with *Ric8a* expression pattern at that developmental stage, since X-gal staining in *Ric8a* ^lacZ/+^ mice was detected in the same areas (Figure 1E). We also mapped the expression of *Ric8aLacZ* and *SynCre* transgene in the newborn mouse brain and detected a very good overlap in several areas of the brain, like in piriform cortex, cornu ammonis 3 (CA3) pyramidal and dentate granule cells of hippocampus (data not shown).

We assessed the deletion of *Ric8a* by analysing genomic DNA isolated from different regions of central nervous system and from some non-neural organs by allele specific PCR. The floxed null allele (300 bp) is generated through the deletion of the intervening DNA sequence situated between *loxP* sites. Deletion had taken place specifically in neural tissue of *Ric8a* ^*CKO*^ mice (in spinal cord and all observed brain regions) and not in non-neural organs (Figure 1C) with the exception of testes (data not shown). In line with this, the *SynCre* activity in testes has also been described earlier [29]. Further, we analysed the completeness of *Ric8a* ablation with *in situ* hybridization, by comparing *Ric8a* expression in *Ric8a* ^*CKO*^ mice and littermate controls using brain sections. We discovered that a substantial amount of *Ric8a* was still transcribed in the brain of *Ric8a* ^*CKO*^ mice. However, its expression pattern was more granular and the overall amount of transcribed *Ric8* was reduced (Figure S1). For negative control, we incubated tissue sections in identical conditions to the sense probe transcript. As a result we did not detect staining in the control tissue (data not shown). In an attempt to quantify the residual expression of *Ric8a*, we used relative qRT-PCR and analysed *Ric8a* level in hippocampus and in the spinal cord using the cardiac muscle and liver as non-neural controls. This analysis clearly demonstrated that *Ric8a* was down regulated in the respective brain regions of *Ric8a* ^*CKO*^ mice (Figure 1F). Furthermore, also the RIC8A protein level was markedly lower in the nervous system of *Ric8a* ^*CKO*^ mice, both in central (hippocampus and spinal cord) and peripheral (spinal ganglia) nervous system (Figure 1G). The level of RIC8A in the tissues used for non-neural control (cardiac muscle, skeletal muscle, liver) was very low in both mutants and littermate controls (Figure 1G and data not shown). These data corroborate that neuron-specific expression of Cre-recombinase results in depletion of *Ric8a* specifically from the nervous system of mouse.

### The conditional ablation of Ric8a results in postnatal death and growth retardation

**Figure 2:**
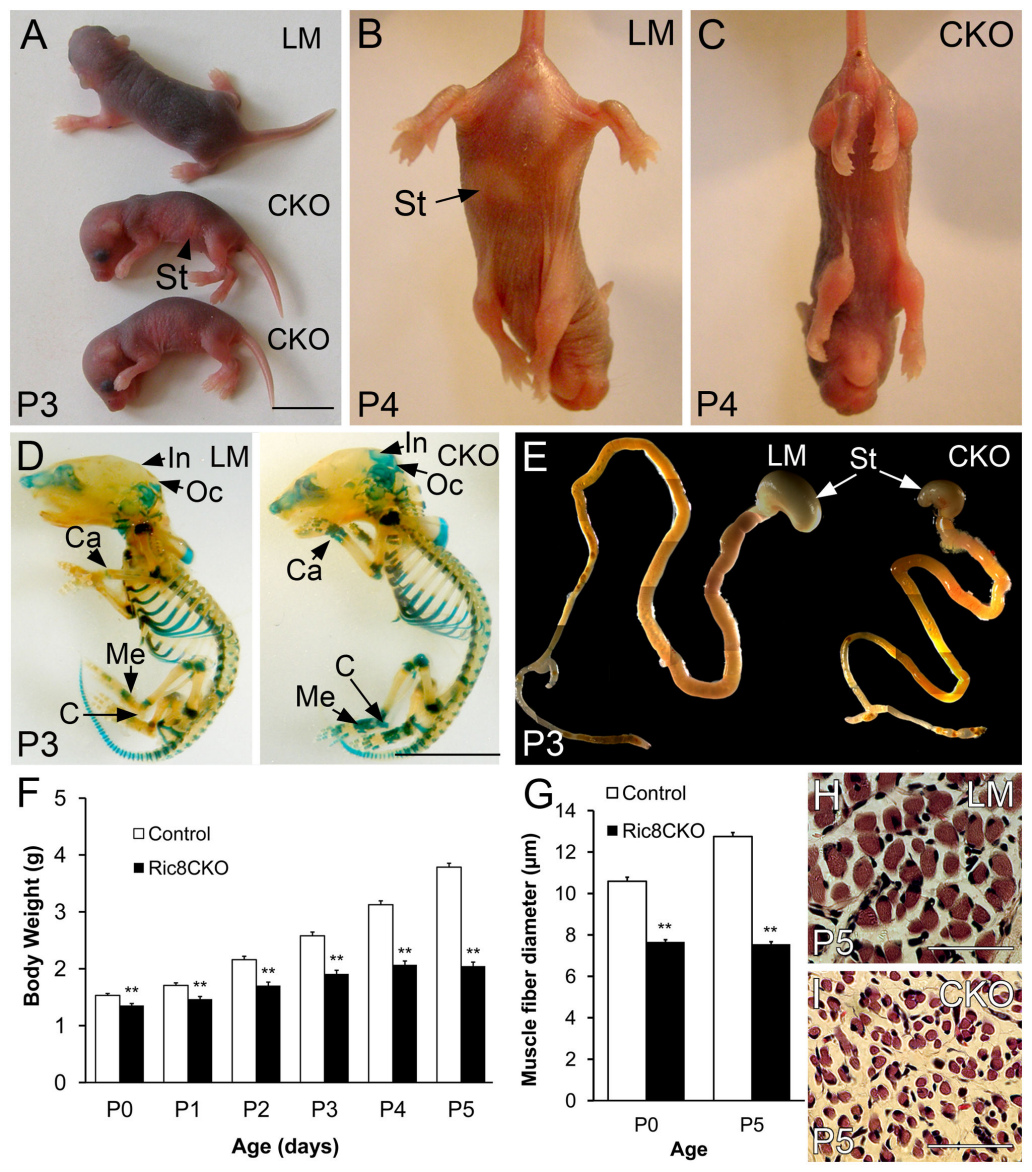
Characterization of *Ric8a* ^*CKO*^ mice phenotype. (**A**) P3 *Ric8a* ^*CKO*^ pups (CKO) reveal growth retardation and an inability to right themselves in comparison to normal littermate control. (**B** and **C**) Characteristic limb positioning of P4 littermate control and *Ric8a* ^*CKO*^ mutant in tail-suspension test. (**D** and **E**) Delayed ossification of *Ric8a* ^*CKO*^ (right) mouse compared to littermate control pup (left) at P3 stage. Cartilage was stained with Alcian Blue. (**F**) Stomach and intestinal tract of *Ric8a* ^*CKO*^ and littermate control (LM) mice. (**G**) Body weight dynamics of *Ric8a* ^*CKO*^ and littermate controls. n(Ric8a^CKO^): P0 = 23, P1 = 20, P2 = 21, P3 = 21, P4 = 13, P5 = 9; n(Control): P0 = 59, P1 = 59, P2 = 59, P3 = 59, P4 = 54, P5 = 42. (**H**) Diameter of skeletal muscle fibres. Histological cross sections of (**I**) littermate control and (**J**) *Ric8a* ^*CKO*^ muscle (n = 3 per genotype per age). Abbreviations: C, calcaneum; Ca, carpal bone; In, interparietal bone; Me, metacarpal bone; Oc, occipital bone; St, stomach. ** *P* < 0,01; Student’s *t* test. Error bars represent mean ± SEM scores. Scale bars: (A, D and E) 1 cm; (I and J) 50 µm.

Neurospecific *Ric8a* conditional mutant mice were born at expected Mendelian ratio (24% of offspring), but died shortly after birth (P0-P6). The majority of analyzed *Ric8a* ^*CKO*^ mice died between P4-P6. Notably, some *Ric8a* ^*CKO*^ pups were abandoned or killed by their mother during a few first days after their birth. Heterozygous littermates (*SynCre* ^*+/-*^ *Ric8a* ^*+/F*^, *SynCre* ^*-/-*^ *Ric8a* ^*+/F*^; *SynCre* ^*-/-*^ *Ric8a* ^*lacZ/F*^), in contrary, were viable and had no apparent morphological deviations (Figure 2A and B). The body weight of *Ric8a* ^*CKO*^ mice was significantly (*P* < 0.01) lower as compared to littermates throughout the observed period (P0-P5; Figure 2G) with no statistically significant gender differences. At birth *Ric8a* ^*CKO*^ mice weighed 1,36 ± 0,03 g which is markedly lower than that of their littermate controls (1,53 ± 0,02 g; Figure 2G). *Ric8a* ^*CKO*^ mice were able to gain weight, but not in exponential manner as their littermates and by P4 they weighed 2,07 ± 0,07 g while their littermates weighed 3,13 ± 0,11 g (Figure 2G). Later, at P5 the weight gain of *Ric8a* ^*CKO*^ mice stopped completely (2,05 ± 0,07 g; Figure 2G), which might be caused by the absence of milk in the stomach compared to littermate controls (Figure 2B and C). Closer examination of stomach of *Ric8a* ^*CKO*^ pups indicated that *Ric8a* ^*CKO*^ mice were initially able to feed, because their stomach contained milk in the early neonatal period. However, starting from P3 onwards, the amount of milk in the stomach decreased (Figure 2F). In order to analyse the maldevelopment of *Ric8a* ^*CKO*^ mutants, we weighed their isolated internal organs separately. The majority of the *Ric8a* ^*CKO*^ organs had lower weight than these from their littermates, because mutant mice are smaller. In general, the ratio of organ mass to body mass fell in three categories: proportional to littermates (heart, kidney, lung), lower than their littermates (liver, spleen) or higher compared to their littermates (brain). Stomach weight was also significantly lower in *Ric8a* ^*CKO*^ mice than in their littermates, but this was probably due to lower amount of milk as described above (data not shown).

Characteristically *Ric8a* ^*CKO*^ pups had hunched back and out of proportion body shape (head was relatively bigger as compared to the rest of the body) (Figure 2A–C). Because of the altered body posture of *Ric8a* ^*CKO*^ mice compared to their littermates, we investigated whether their skeleton had formed correctly. We did not find any structural abnormalities or loss of skeletal elements. However, Alcian Blue staining revealed an ossification delay (e.g. in carpal and metacarpal bones, in interparietal and occipital bones) in P3 *Ric8a* ^*CKO*^ mice (Figure 2D and E), but no difference was observed at P0 stage (data not shown). Taken together, these results strongly suggest that GEF RIC8A is necessary in differentiated neurons for survival as well as for the correct postnatal development.

### Ric8a^CKO^ mice showed severe neurological impairment

*Ric8a* ^*CKO*^ mice have several neuromuscular defects. They are hypoactive, exhibit general ataxia and have spontaneous convulsions and spasms (Video S1). In an attempt to evaluate the neuromotor performance of *Ric8a* ^*CKO*^ pups we performed simple handling assays. First of all, we observed that *Ric8a* ^*CKO*^ mice always lied on their sides and were not able to fully right themselves (Figure 2A; Video S2). Secondly, to monitor neurological reflexes, we tested the “pinching response” by pinching the tail or limbs of the newborns. *Ric8a* ^*CKO*^ mutants reacted adequately by flailing their limbs trying to change their body position demonstrating that the tactile response was not lost. Moreover, pain reflex did not seem to be altered. As a third neuromotor assay, we performed a tail-suspension test. Control littermates spread their limbs whereas *Ric8a* ^*CKO*^ mice remained almost completely immobile only moving their forelimbs in an uncoordinated manner (Figure 2B and C). In summary, *Ric8a* ^*CKO*^ mice had severe motor skill deficits and neurobehavioral development abnormalities characterized by hypotonia.

### Deficiency of RIC8A in neurons leads to skeletal muscle atrophy in mice

The phenotype analysis clearly indicated the evident neuromuscular defects in *Ric8a* ^*CKO*^ mice. Furthermore, the histological analysis of skeletal muscles at P0 and P5 revealed that the skeletal muscle tissue in *Ric8a* ^*CKO*^ pups was hypoplastic compared to littermate controls. This is probably caused by the atrophy of myocytes, because we did not find a difference in the number of muscle fibres, whereas the diameter of the muscle fibres was markedly decreased. Measurement of muscle fibre diameters in the cross sections revealed that the diameter of *Ric8a* ^*CKO*^ muscle cells was significantly (*P* < 0.01) smaller compared to littermate control (Figure 2H; n = 3 per genotype per age). The myofibres of *Ric8a* ^*CKO*^ mice were also sparsely distributed and less compact than in their littermates (Figure 2I and J). The analysis of skeletal muscle morphology demonstrates that the ablation of RIC8A function in neurons causes skeletal muscle atrophy. We presume that the reason for muscle atrophy is an insufficient neuronal stimulation at the neuromuscular junction.

### Ablation of RIC8A in differentiated neurons does not cause changes in gross brain morphology

**Figure 3:**
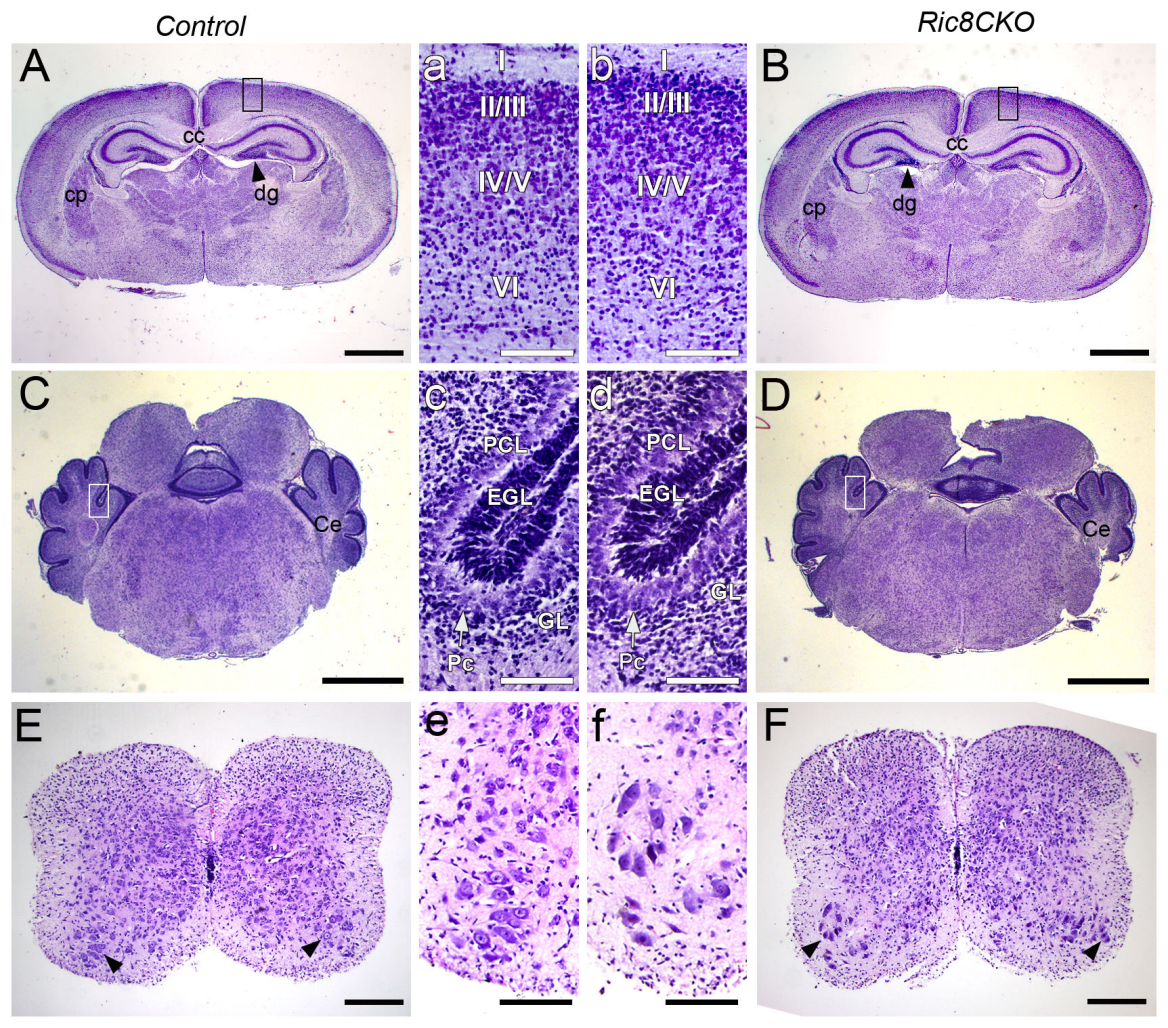
Histological analysis of *Ric8a* ^*CKO*^ mutants brains and comparison to littermate control mice. (**A**–**D**) Representative images of hematoxylin-eosin stained P3 coronal brain sections. No gross difference was detected in *Ric8a* ^*CKO*^ brain. (**A** and **B**) Anterior sections of neonatal brains. (**a** and **b**) Magnified image of neocortex layers from black boxes in A and B respectively. (**C** and **D**) Posterior part of neonatal brains. (**c** and **d**) Magnified image of cerebellar cortex layers from white boxes in C and D respectively. (**E** and **F**) Representative spinal cord sections from thoracic region. Motor neurons are indicated with black arrowheads. (**e** and **f**) Magnified image of ventral horns from E and F respectively. Abbreviations: cc, *corpus callosum*; Ce, cerebellum; cp, *caudate putamen*; dg, dentate gyrus; EGL, external granular layer; GL, granular layer; Pc, Purkinje cell; PCL, Purkinje cell layer; I, II/III, IV/V, VI, neocortical cell layers. Scale bars: (A-D) 1 mm; (E and F) 200 µm; (a-f) 100 µm.

Brains of *Ric8a* ^*CKO*^ mice were about the same size as the brains of littermate controls. As compared to body mass the brains of *Ric8a* ^*CKO*^ mutants were relatively bigger, which indicates that although *Ric8a* ^*CKO*^ mice experience growth retardation, their brain development has not been disturbed. In order to characterise the possible developmental abnormalities in central nervous system of *Ric8a* ^*CKO*^ mice, we performed a systematic histological analysis. Examination of hematoxylin-eosin stained coronal sections of *Ric8a* ^*CKO*^ (Figure 3B and b; Figure 3D and d) and littermate brains (Figure 3A and a; Figure 3C and c) and spinal cords (Figure 3E and e; Figure 3F and f) revealed no major differences either in the size or morphology of main structures. Moreover, we also counted motor neurons, because it has been shown that the mice with neuromuscular defects have a reduced number of motor neurons [30,31], but we did not find a significant difference between *Ric8a* ^*CKO*^ mice and littermate controls: the average number of motor neurons per section was 17,7 ± 0,7 and 18,8 ± 0,7 respectively (n = 3 per genotype). The above mentioned results demonstrate that the aberrant phenotype of *Ric8a* ^*CKO*^ mutants is not caused by gross morphological anomalies of the central nervous system.

### Deficiency of RIC8A in neurons causes heart muscle hypoplasia and abnormal morphology of the sinoatrial node

**Figure 4:**
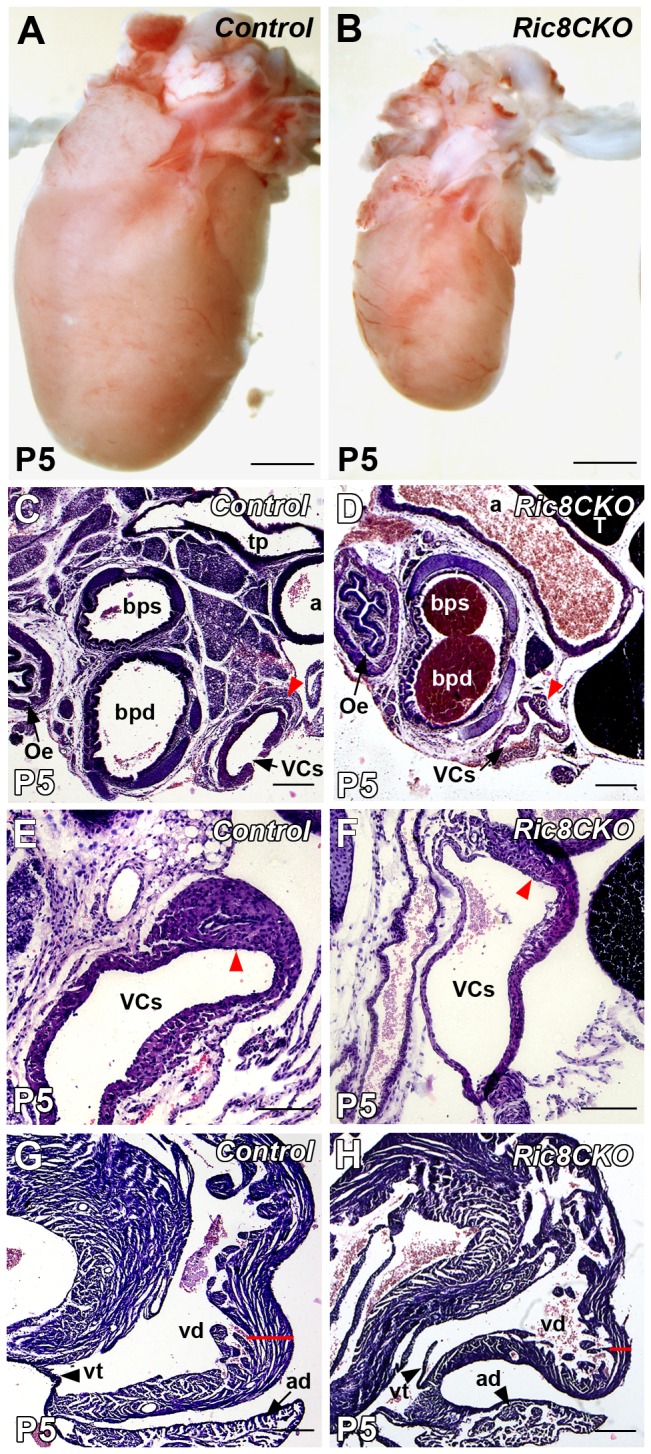
Histological analysis of the heart of *Ric8a* ^*CKO*^ mutant mice compared to littermate control. (**A** and **B**) Representative images of hearts of P5 mice. (**B**) Heart of *Ric8a* ^*CKO*^ mouse is markedly smaller. (**C**–**H**) Anterior-most part of the sinoatrial node extends to the branching level of main bronchi and to the level of transverse aortic arch. (**C** and **D**) The beginning of sinoatrial node (indicated by red arrowhead) is located more anteriorly in the heart of *Ric8a* ^*CKO*^ mutant mice. We used separated bronchi (bps, bpd) and aorta (a) as reference. (**E** and **F**) The medio-ventral wall of superior vena cava (VCs) containing sinoatrial node (red arrowhead) is thinner in *Ric8a* ^*CKO*^ mice compared to littermates. (**G** and **H**) The wall of the right ventricle (vd) is thinner and has less trabeculi in *Ric8a* ^*CKO*^ heart (indicated by red line). Abbreviations: a, aorta; ad, *atrium dextrum*; bpd, *bronchus principalis dexter*; bps, *bronchus principalis sinister*; Oe, oesophagus; T, thymus; tp, *truncus pulmonalis*; VCs, superior *vena cava* ; vd, *ventriculus dexter*; vt, *valva tricuspidalis*. Scale bars: (A, B) 1 mm; (C,D, G, H) 200 µm; (E and F) 100 µm.

To further detail the neuromuscular phenotype of *Ric8a* ^*CKO*^ mice, we assessed their hearts as the secondary indicators of the defects of peripheral nervous system. Inspection of different organs in *Ric8a* ^*CKO*^ mice revealed that their hearts were markedly smaller than the hearts of their littermate controls (Figure 4A and B). Although dissected hearts usually pump all the blood out, the analysis of hematoxylin-eosin stained sections revealed that dissected *Ric8a* ^*CKO*^ hearts contained much more blood than hearts of littermates (Figure 4C and D). Further, we focused on the sinoatrial node, since the changes in its morphology were the most prominent at first glance. Sinoatrial node is innervated by both sympathetic and parasympathetic axons, and it contains pacemaker cells, which are responsible for the generation of sinus rhythm. The sinoatrial node of *Ric8a* ^*CKO*^ mice was located more anteriorly than in littermates. The anterior-most part of sinoatrial node extended to the branching level of main bronchi and aortic arch is visible in the cross-section (Figure 4D). The anterior-most part of the littermate sinoatrial node in contrary extended to the level where trachea has been already divided into main bronchi (Figure 4C). Sinoatrial node is located in the medio-ventral wall of superior *vena cava*, which was significantly thinner in *Ric8a* ^*CKO*^ mutant mice (Figure 4F) compared to control littermate (Figure 4E). Hence, the sinoatrial node itself appeared to be substantially smaller (Figure 4E and F) and the myocardium of *Ric8a* ^*CKO*^ mice was markedly thinner than in littermates (Figure 4G and H). The substantially different size of myocardium was also apparent at inspection of intact hearts (Figure 4A and B).

The cardiac function was further assessed by recording the heart rates of P1 *Ric8a* ^*CKO*^ mice and littermate controls using electrocardiography (ECG) analysis. The heart rate of *Ric8a* ^*CKO*^ mice was significantly (*P* < 0,05) slower than that of their littermates: 269,2 ± 6,2 beats per minute in *Ric8a* ^*CKO*^ mice (n = 5), and 327,7 ± 25,3 in the littermate control (n = 3), respectively. In addition, by analysing the ECG recordings we also discovered that *Ric8a* ^*CKO*^ mice experience skeletal muscle spasms, some at regular intervals. Furthermore, the heart rate of *Ric8a* ^*CKO*^ mice did not respond as effectively to the skeletal muscle activity during voluntary movements as in controls (data not shown). These results indicate that in parallel with the skeletal muscle atrophy, *Ric8a* ^*CKO*^ mice have strong cardiac muscle hypoplasia, their sinoatrial node is underdeveloped and the cardiac function impaired.

## Discussion

In the current study we created mutant mice with RIC8A specifically knocked out from differentiated neurons in order to elucidate the physiological function of RIC8A in the nervous system and to circumvent the embryonic lethality of Ric8a^-/-^ mice. The main goal was to analyse the phenotype of the developed *Ric8a* ^*CKO*^ mutants and therefore provide an insight into the role of RIC8A in the nervous system *in vivo*. The deficiency of RIC8A was confirmed by using genomic PCR, quantitative RT-PCR and western blot analysis. Our data corroborated the authenticity of neurospecific *Ric8a* ^*CKO*^ line and thus the severe phenotype observed in the *Ric8a* ^*CKO*^ mice can be unambiguously attributed to RIC8A ablation in neurons.

*Ric8a* ^*CKO*^ mutants were born at expected Mendelian ratio but died before P6. The main reason for the early postnatal death is probably malnutrition. Although *Ric8a* ^*CKO*^ mice were initially nursed, their stomach was empty when death occurred. We also found a number of secondary features that indicate malnourishment of mutant mice. First, the bones of *Ric8a* ^*CKO*^ mice at P3 stained more extensively for cartilage than in control mice, which means that ossification was impaired, since at birth the bones of mutant mice revealed no apparent delay in ossification. The result is consistent with the fact that postnatal nutrition and bone development are known to be directly linked [32,33]. Second, the liver of *Ric8a* ^*CKO*^ mutants weighed significantly less in relation to body mass compared to the liver of littermate controls. The brain weight of *Ric8a* ^*CKO*^ mice, in contrary, was almost the same as of littermate controls, which means that in relation to body mass the brains of mutant mice were significantly heavier than in control mice. The brain to liver weight ratio is usually considered as an indicator of malnutrition, because brain development is a priority and it takes place at the expense of somatic tissues [34]. As mentioned, the brain to liver weight ratio was significantly higher in mutant mice confirming their malnutrition. The phenotypic appearance of mouse newborns presenting similar neuromuscular defects often die neonatally due to a respiratory failure and their lifespan is usually less than 24 hours [35–37]. Since *Ric8a* ^*CKO*^ mice were able to survive until P6 it is unlikely that their death is caused by a respiratory failure. Therefore, it is most likely that the *Ric8a* ^*CKO*^ mice died because of malnutrition and the resulting dehydration. *Ric8a* ^*CKO*^ mice are capable of sucking mother’s milk, but perhaps since their locomotion is impaired, they cannot compete with their littermates, when latter develop better motor skills.

The deficiency of RIC8A in neurons resulted in a severe neuromuscular phenotype in *Ric8a* ^*CKO*^ mice, characterized by abnormal body curvature, inability to right themselves, muscular spasms and impaired motor skills. RIC8A functions as a receptor-independent activator for Gα_i_, Gα_q_, Gα_o_ and Gα_13_ subunit families [2]. This particular activity has been confirmed by separate studies that demonstrate the capacity of RIC8A to potentiate the signal of Gα_q_ [19] and Gα_i_ [38,39]. Recently, RIC8A was suggested to function as a molecular chaperone required for Gα subunit biosynthesis [17]. In addition, a very recent study showed that human neural cell adhesion molecule NCAM180 potentiates the Gα_s_ coupled β-adrenergic receptor response in a RIC8A dependent manner [40]. The collective data on the biochemical function of RIC8A protein strongly suggest that the neuromuscular defects of *Ric8a* ^*CKO*^ mice are caused by reduced activity of G proteins in neurons. Thus, different Gα subunit knockout mice should partly recapitulate the *Ric8a* ^*CKO*^ phenotype. Indeed, we found some similarities with *Gα*_*o*_^*-/-*^ mice. Gα_o_ is highly expressed in neurons and mediates effects of a group of rhodopsin-like receptors that include the opioid, α2-adrenergic, M2 muscarinic and somatostatin receptors. *Gα*_*o*_^*-/-*^ mice had an average half-life of 7 weeks, they were weaker and smaller and had impaired motor control [41]. Furthermore, *Gα*_*q*_^*-/-*^ mice also displayed neural phenotype – they had ataxia and impaired motor control [42]. Since G protein mediated signals result in wide variety of cellular and systemic effects, it is difficult to pinpoint the main trigger or a mechanism for the neuromuscular phenotype of *Ric8a* ^*CKO*^ mice.

The phenotype of *Ric8a* ^*CKO*^ mice cannot be attributed to insufficient differentiation or malformation of the central nervous system. The histological examination and counting of cells in spinal cord of *Ric8a* ^*CKO*^ mutant mice revealed no gross differences. In comparison with other organs, the brain weight of mutant mice showed the lowest lag in development. The obtained results are consistent with the transgenic nature of *Ric8a* ^*CKO*^ mice, since RIC8A is only absent from differentiated neurons and its function in other cells or in division and migratory processes should not be affected. Our *in situ* hybridization results indicate that *Ric8a* is still extensively transcribed in the brains of *Ric8a* ^*CKO*^ mice, although the mRNA expression tends to be more granular than in control mice. The change in expression pattern was particularly prominent in the hippocampus, being surprisingly higher in dentate gyrus and in CA3, but lower in CA1 as compared to littermates. This contradicts the *SynCre* expression pattern as SynCre has been shown to be highly active in dentate gyrus and CA3 region [25]; thus *Ric8a* expression is expected to be lower in these regions in *Ric8a* ^*CKO*^ mice but not higher as was detected with the *in situ* hybridization analysis. However, the relative *Ric8a* mRNA expression and also RIC8A protein level in the hippocampus of *Ric8a* ^*CKO*^ mice clearly show the strong downregulation of RIC8 is. Most probably, the expression is detected in the hippocampus of mutant mice is derived from glial cells, where it could be upregulated due to signals received from the neighbouring RIC8 deficient neurons. Consistent with that, it was recently demonstrated that RIC8 is required in glial cells, specifically in the Bergmann glia during cerebellar foliation [43]. *Ric8a in situ* hybridization pattern might also be due to the specificity of *SynCre* expression that only eliminates *Ric8a* in differentiated neurons [23], but since neurogenesis in dentate gyrus continues postnatally [44], *Ric8a* is still present in these dividing cells. It is highly probable that *Ric8a* ^*CKO*^ mice would develop more apparent neurological abnormalities if they could live longer and develop further.

*SynCre* is expressed in the peripheral nervous system in addition to the central nervous system and we confirmed the deficiency of RIC8A in peripheral nervous system by western blot of synaptic ganglia. This implies that RIC8A is absent in neurons that control the heart function, breathing, motor function and other vital processes. Consistently, we found that *Ric8a* ^*CKO*^ mice had skeletal muscle atrophy, which was probably due to insufficient signalling at the neuromuscular junctions. Since *Ric8a* ^*CKO*^ mice lied on their sides and did not move around, their muscles fell even more behind in development, which resulted in more severe atrophy. Furthermore, we found that *Ric8a* ^*CKO*^ mutant mice had heart muscle hypoplasia. Moreover, the heart of *Ric8a* ^*CKO*^ mice contained significantly more blood compared to the heart of littermates, which could indicate malfunctioning of the cardiac muscle, because heart could not pump out blood after dissection. In addition we found that the sinoatrial node of *Ric8a* ^*CKO*^ mice was displaced and smaller compared to the littermate controls. Sinoatrial node is a pacemaker tissue in the right atrium of the heart that is responsible for the generation of normal sinus rhythm. When sinoatrial node is defective, the heart rhythm becomes abnormal – either too fast, too slow, or their combination [45]. The heartbeat of *Ric8a* ^*CKO*^ mutants was slower than of littermate controls but rhythmical. Disruption in the structure of the ECG complexes or abnormalities in the rhythm were not found in each individual mutant mouse. Although muscular activity demands a change in heart rhythm, the heart rate of mutant mice did not respond in similar magnitude compared to littermates during their movements while ECG was recorded, indicating dysfunction in general coordination of autonomic nervous system. *Ric8a* ^*CKO*^ mice also experienced muscular spasms at regular intervals. Since RIC8A is absent only in neurons but not in muscle tissue, the postsynaptic muscle tissue should be competent for the signal reception. Therefore, the anomalies detected in heart and skeletal muscle tissue should primarily be the result of impaired functioning of signalling neurons. Taken together, our data emphasize the essentiality of RIC8A for the proper functioning of the nervous system.

In summary, here we provide novel data about the critical role of RIC8A in the mammalian nervous system. By using conditional ablation we were able to assess the function of RIC8A in differentiated neurons only, demonstrating that the ablation of RIC8A in neurons results in severe neuromuscular phenotype and early postnatal death of mice. Further studies are needed to elucidate the exact mechanism of RIC8A in neuronal signalling in mammals. 

## Supporting Information

Video S1 ***Ric8A*^*CKO*^ mice are exhibit general ataxia and have spontaneous convulsions and spasms.** One P3 *Ric8A* ^*CKO*^ mutant mouse is shown.
(MP4)

Video S2 ***Ric8A*^*CKO*^ mice are hypoactive and unable to right themselves.** Two P3 *Ric8A* ^*CKO*^ mutant mice and one littermate control (in the middle) are shown.
(MP4)

Figure S1 **Analyses of *Ric8* expression in mouse brains by in situ hybridization.** Compared to littermate controls (A-C) the expression pattern has changed in *Ric8A* ^*CKO*^ and the overall amount of transcribed *Ric8* has reduced (D-F). Abbreviations: CA1/CA3, *Cornu ammonis* regions 1 and 3; CC, corpus callosum; DG, dentate gyrus; I, II/III, IV/V, VI, neocortical cell layers. Scale bars (representative of all images): (A-D) 500 µm.
(TIF)
