# Supplementary figures and images for: Ablation of RIC8A Function in Mouse Neurons Leads to a Severe Neuromuscular Phenotype and Postnatal Death
Source: PLoS One. 2013 Aug 16;8(8):e74031. doi: 10.1371/journal.pone.0074031 (PMC3745415; doi:10.1371/journal.pone.0074031)

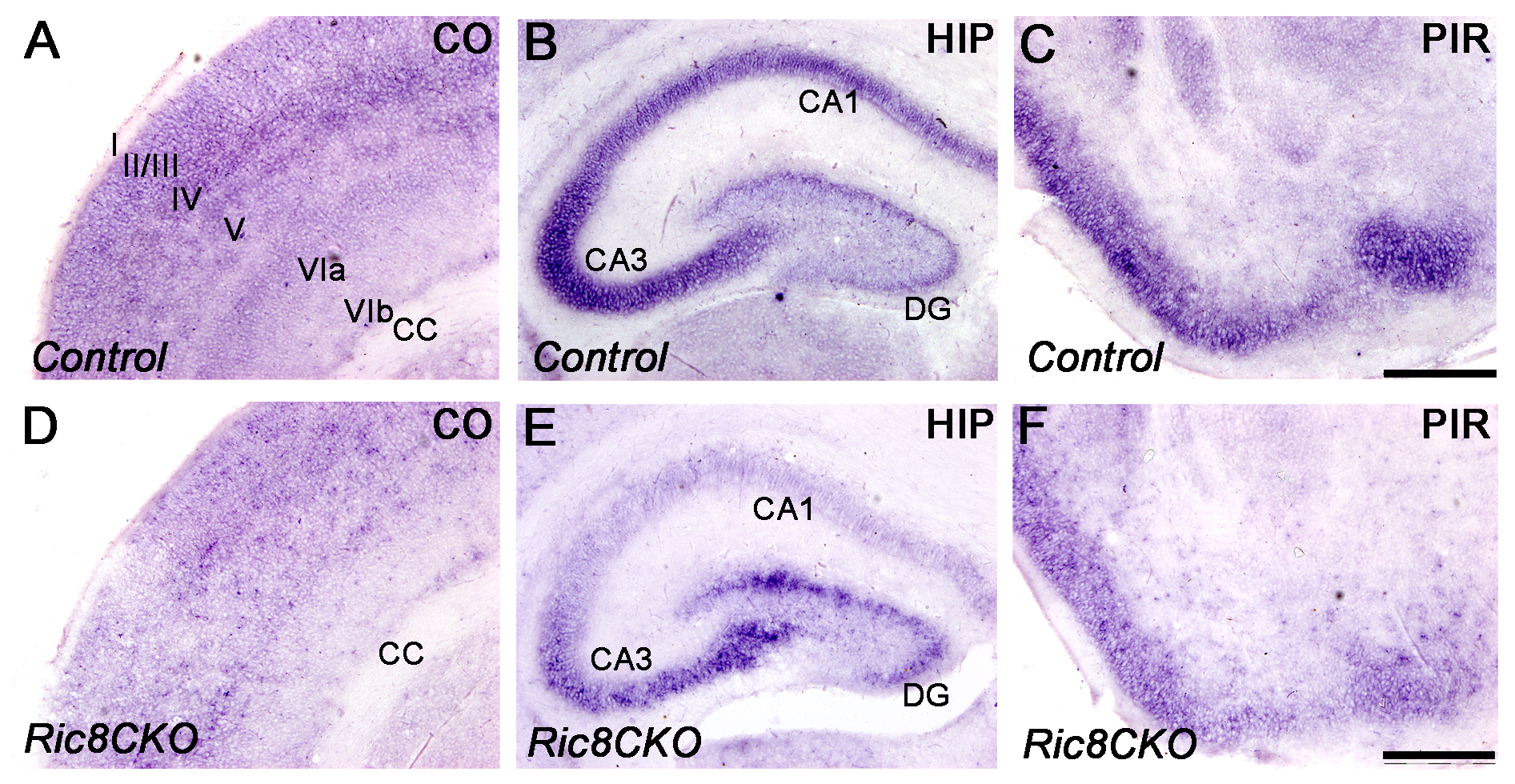

Supplement: Figure S1 — Analyses of Ric8 expression in mouse brains by in situ hybridization. Compared to littermate controls (A-C) the expression pattern has changed in Ric8A CKO and the overall amount of transcribed Ric8 has reduced (D-F). Abbreviations: CA1/CA3, Cornu ammonis regions 1 and 3; CC, corpus callosum; DG, dentate gyrus; I, II/III, IV/V, VI, neocortical cell layers. Scale bars (representative of all images): (A-D) 500 µm. (TIF) [file pone.0074031.s003.tif]
